# Distinct Contributions of Rod, Cone, and Melanopsin Photoreceptors to Encoding Irradiance

**DOI:** 10.1016/j.neuron.2010.04.037

**Published:** 2010-05-13

**Authors:** Gurprit S. Lall, Victoria L. Revell, Hiroshi Momiji, Jazi Al Enezi, Cara M. Altimus, Ali D. Güler, Carlos Aguilar, Morven A. Cameron, Susan Allender, Mark W. Hankins, Robert J. Lucas

**Affiliations:** 1 Faculty of Life Sciences, AV Hill Building, University of Manchester, Manchester M13 9PT, UK; 2 Department of Computer Science, Regent Court, 211 Portobello, University of Sheffield, Sheffield S1 4DP, UK; 3 Departments of Biology and Neuroscience (JHMI), 227 Mudd Hall/3400 North Charles Street, Johns Hopkins University, Baltimore, MD 21218, USA; 4 Nuffield Laboratory of Ophthalmology, University of Oxford, John Radcliffe Hospital, Oxford OX3 9DU, UK

**Keywords:** SYSNEURO, CELLBIO

## Abstract

Photoreceptive, melanopsin-expressing retinal ganglion cells (mRGCs) encode ambient light (irradiance) for the circadian clock, the pupillomotor system, and other influential behavioral/physiological responses. mRGCs are activated both by their intrinsic phototransduction cascade and by the rods and cones. However, the individual contribution of each photoreceptor class to irradiance responses remains unclear. We address this deficit using mice expressing human red cone opsin, in which rod-, cone-, and melanopsin-dependent responses can be identified by their distinct spectral sensitivity. Our data reveal an unexpectedly important role for rods. These photoreceptors define circadian responses at very dim “scotopic” light levels but also at irradiances at which pattern vision relies heavily on cones. By contrast, cone input to irradiance responses dissipates following light adaptation to the extent that these receptors make a very limited contribution to circadian and pupillary light responses under these conditions. Our data provide new insight into retinal circuitry upstream of mRGCs and optimal stimuli for eliciting irradiance responses.

## Highlights

► Red cone knockin (*Opn1mw^R^*) mice reveal rod, cone, and melanopsin phases to NIF vision ► Rods drive circadian responses to very low irradiances (scotopic threshold) ► Circadian responses to light in the photopic range can be rod driven ► Light adaptation limits the influence of cones on NIF vision

## Introduction

The mammalian eye extracts at least two fundamentally different sorts of information from the light environment. Its most widely appreciated role is to provide a spatial map of the visual scene. However, equally important is its ability to encode irradiance, the ambient level of light. This irradiance information defines a variety of nonimage forming (NIF) behavioral and physiological responses to light. These include synchronization of endogenous circadian clocks to light:dark (LD) cycles (photoentrainment) and regulation of pupil size, pineal melatonin production, sleep propensity, and aspects of gross physiology.

Recent years have seen the discovery and elucidation of a new class of retinal photoreceptor seemingly dedicated to irradiance detection. This comprises a small number of directly photosensitive retinal ganglion cells (Berson et al., 2002) expressing melanopsin (mRGCs), a retinaldehyde-based photopigment (Lucas et al., 2003; Melyan et al., 2005; Panda et al., 2005; Qiu et al., 2005). These mRGCs innervate brain nuclei involved in NIF vision, and selective ablation of this ganglion cell class abolishes irradiance responses in mice (Göz et al., 2008; Güler et al., 2008; Hatori et al., 2008).

The presence of mRGCs would appear to free the retina's most abundant photoreceptors (rods and cones) from contributing to irradiance pathways. However, mRGCs receive synaptic input from the outer retina (Belenky et al., 2003; Dacey et al., 2005; Perez-Leon et al., 2006; Viney et al., 2007; Wong et al., 2007) and, while circadian and pupillary responses are retained in mice lacking rods and cones (Freedman et al., 1999; Lucas et al., 1999, 2001), they also survive melanopsin knockout (Lucas et al., 2003; Panda et al., 2002; Ruby et al., 2002). Because mice lacking all three receptor types lose all responses (Hattar et al., 2003; Panda et al., 2003), it seems that NIF vision can be supported by photoreception in either rods and cones or melanopsin.

The discovery of mRGCs has seen an application in the field of lighting design, with evidence that brighter and/or “bluer” lights can improve health and well-being for a number of at risk populations in both clinical and field settings (Riemersma-van der Lek et al., 2008; Viola et al., 2008). One of the barriers to optimizing these strategies is uncertainty regarding the contribution of rods and cones to mRGC-driven behaviors. To date, the most abundant data on this subject comes from studies of mice carrying genetic lesions of either rod and cone or melanopsin photoreception. Much of it supports the view that melanopsin provides a specific high-irradiance signal while rods/cones drive responses to dimmer light (Lucas et al., 2001, 2003; Mrosovsky, 2003; Panda et al., 2003). This hypothesis is consistent with physiological recordings from mRGCs (Dacey et al., 2005; Schmidt et al., 2008; Wong et al., 2007). However, in vivo data is only partly consistent with this model because, for example, mice lacking rods and cones do not always show the predicted decrease in sensitivity (Freedman et al., 1999; Lucas et al., 1999; Lupi et al., 2008), whereas deficits in circadian photoresponsiveness in melanopsin knockout mice extend to dim intensities (Panda et al., 2002). Moreover, some reports have suggested that NIF responses can be entirely dependent upon melanopsin without detectable rod or cone contributions (Lupi et al., 2008; but see also Altimus et al., 2008; Tsai et al., 2009). It is unclear the extent to which these inconsistencies reflect compensatory/disruptive reorganization associated with the relatively invasive methods used to isolate rod, cone, and melanopsin responses (retinal degeneration and/or gene knockout), or genuine plasticity in the contribution of the three photoreceptor types.

The most satisfactory approach to resolving these discrepancies and generating a more complete picture of irradiance coding to include the separate role of rods and cones would be to determine the contribution of each photoreceptor to irradiance responses without having to compromise the functional integrity of the retina. Here we achieve this goal using transgenic mice (*Opn1mw^R^*) carrying a human red cone opsin coding sequence in place of their native m-cone opsin (Smallwood et al., 2003). These animals display a long-wavelength shift in cone spectral sensitivity, sufficient for cone-dependent activity (and indeed that of rods and melanopsin) to be independently identified by comparing sensitivity to a medium versus a long wavelength light. Our description of circadian and pupillary responses in this mouse reveals largely nonoverlapping rod, cone, and melanopsin phases to NIF vision, defined according to stimulus irradiance and the pattern of prior light exposure.

## Results

### Regulation of the Dark-Adapted Pupil by Cones and Melanopsin

**Figure 1:**
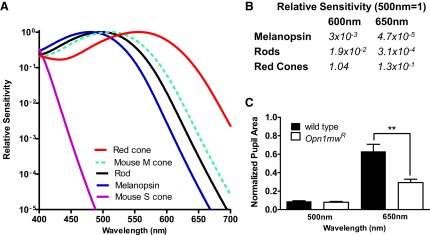
Enhanced Long-Wavelength Sensitivity of Cone Vision in *Opn1mw^R^* Mice (A) In *Opn1mw^R^* mice, the native mouse m-cone opsin (dotted green line, shows spectral sensitivity approximated by opsin nomogram [Govardovskii et al., 2000] with peak sensitivity [λ_max_] = 511 nm) is lost and replaced with a human red cone opsin (Smallwood et al., 2003) whose spectral sensitivity (red line; λ_max_ = 556 nm) profile is quite distinct from that of mouse rod (λ_max_ = 498 nm), melanopsin (λ_max_ = 480 nm), and s-cone (λ_max_ = 360 nm) opsins (black, blue, and purple lines, respectively). (B) Divergence in the spectral sensitivity of red cones, rods, and melanopsin is reflected in large differences in their relative sensitivity to mid- (500 nm) and long- (600 or 650 nm) wavelength light. (C) Red cone input to the pupil light reflex is revealed as a significant increase in response to a 1 min, 650 nm stimulus (3 × 10^14^ photons/cm^2^/s) in *Opn1mw^R^* mice compared with wild-type mice (mean ± SEM; t test, p < 0.01). The genotypes showed similar responses to an equivalent (1 min; 3 × 10^14^ photons/cm^2^/s) 500 nm stimulus (mean ± SEM; n = 8–12; t test, p > 0.05).

The red cone knockin allele (referred to here as *Opn1mw^R^*) results in a substantial, long-wavelength shift in the spectral sensitivity of those cones that would ordinarily express m-cone opsin (Figure 1; Smallwood et al., 2003). Electrophysiological and behavioral assessments suggest that these red cones retain normal function and retinal connectivity (Jacobs et al., 2007; Smallwood et al., 2003). To determine whether they are capable of driving NIF responses, we first compared pupillary responses to bright medium- (500 nm) and long-wavelength (650 nm) stimuli in *Opn1mw^R^* males with those of littermate wild-type mice. As expected, the two genotypes had equivalent responses to 500 nm (Figure 1C). However, 650 nm induced much larger constriction in *Opn1mw^R^* animals (Figure 1C), suggesting involvement of cones in this response.

**Figure 2:**
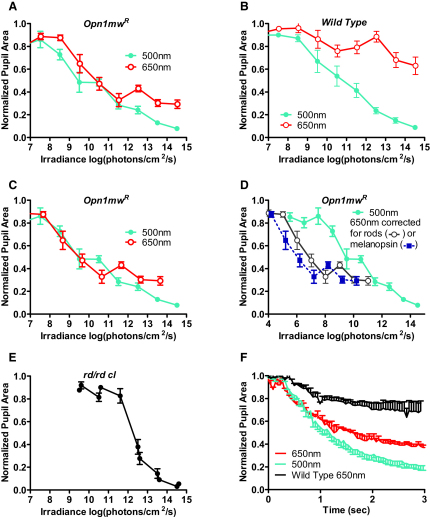
Cone Contributions to Defining Pupil Size (A) Sixty-second stimuli drove irradiance-dependent decreases in pupil size at both 500 and 650 nm in dark-adapted *Opn1mw^R^* mice (n = 4–12). (B) Wild-type mice also responded to both wavelengths, but were much less sensitive to 650 than 500 nm (n = 4–5). (C) Following correction of the 650 nm data from *Opn1mw^R^* mice to allow for the reduced sensitivity of cones to this wavelength versus 500 nm (irradiance of 650 nm stimuli × 0.13), the two irradiance response curves are superimposed at <10^11^ photons/cm^2^/s. F-test analysis reveals that sigmoidal curves fitted to these data sets differ (p < 0.01) in the best fit for their lower asymptote (nonoverlapping 95% confidence intervals), but not in other parameters. (D) There was no such convergence when irradiances were normalized according to the spectral sensitivity of rods or melanopsin. (E) As previously reported (Lucas et al., 2003), the pupillary responses of dark-adapted *rd/rd cl* mice elicited by 60 s 500 nm stimuli had a high threshold (n = 4–5). (F) A detailed examination of pupil size (n = 4) over the first 3 s of exposure (lights on at time = 0) to bright stimuli at 500 nm (2.2 × 10^13^ photons/cm^2^/s) and 650 nm (1.6 × 10^14^ photons/cm^2^/s) isoluminant for red cones revealed that, whereas the early rate of constriction was equivalent, responses at the two wavelengths diverged after ∼0.8 s. The response of wild-type mice to the longer wavelength was smaller and slower, confirming that the 650 nm response over these timescales relies on red cones. All data points show mean ± SEM pupil area normalized to prestimulus condition.

To confirm this finding and to provide a more detailed picture of cone contributions to defining pupil size, we next described full irradiance response relationships at 500 and 650 nm for the dark-adapted pupillary light reflex (PLR). In *Opn1mw^R^* mice there is a substantial divergence in relative sensitivity of melanopsin, rods, and red cones between these two wavelengths (Figure 1B). We found that irradiance response curves at 500 and 650 nm were remarkably similar for *Opn1mw^R^*, but not littermate wild-type, mice (Figures 2A and 2B). In fact, when corrected for the difference in red cone sensitivity at these two wavelengths (650 nm irradiance measures ×0.13), pupil responses became indistinguishable at irradiances <10^11^ photons/cm^2^/s in *Opn1mw^R^* mice (Figure 2C). By contrast, responses were highly divergent when correction factors based upon the relative sensitivity of either rods or melanopsin were applied (Figure 2D). This suggests that under these conditions red cones define the magnitude of pupil constriction in the range of ∼10^8^–10^11^ photons/cm^2^/s. The lower limit of this range is ∼1 log unit below the reported threshold for cone-based vision in mice (Nathan et al., 2006), indicating that cone pathways presynaptic to mRGCs are at least as sensitive as those subserving pattern vision. The upper limit is probably defined by cone saturation, which similarly occurs around 3 log units above threshold for light steps in mice (Nikonov et al., 2006).

The deficiency in responses to 650 nm stimuli >10^12^ photons/cm^2^/s phenocopies the loss of bright-light responses in melanopsin knockout mice (Lucas et al., 2003; Van Gelder et al., 2003). Thus, it seems likely that melanopsin is responsible for encoding these higher irradiances thanks to its sensory specializations for this task (Berson et al., 2002; Do et al., 2009; Zhu et al., 2007). In support of this hypothesis, parallel experiments revealed that rodless and coneless (*rd/rd cl*) mice failed to respond to even the brightest 650 nm stimuli (not shown), while their threshold for a measurable pupillary response at 500 nm was, as previously reported (Lucas et al., 2001), around 10^12^ photons/cm^2^/s (Figure 2E).

Together these data reveal that, under these conditions, the combined action of cones and melanopsin can drive graded decreases in pupil size over at least 6 decimal orders (∼10^8^ to ∼10^15^ photons/cm^2^/s) of irradiance. This impressive dynamic range cannot be explained by a simple linear summation of cone- and melanopsin- dependent responses. Rather it implies a “winner takes all” arrangement in which cones define responses at irradiances below melanopsin threshold, but have little impact on pupil size once melanopsin is activated. Figure S2 (available online) shows one method by which this can be achieved.

### Cones Contribute Response Speed to the PLR

The melanopsin photoresponse is thought to be much slower than that of cones (Berson et al., 2002). To determine whether this temporal separation was reflected in a contribution of these two photoreceptors to the murine PLR, we compared response time courses to 500 nm and 650 nm stimuli matched for their effects on cones (Figure 2F). We found that, as predicted for a response driven by cones, pupil size under the two wavelengths was indistinguishable at short response times (≤0.8 s). However, the contribution of melanopsin became clear at longer time points as responses to 500 nm (at which melanopsin is active) became larger than those at 650 nm. The divergence point for the two curves (around 0.8 s) is similar to previous estimates of the inherent response latency of melanopsin (Lucas et al., 2001).

### Cones Do Not Ordinarily Support Circadian Photoentrainment

**Figure 3:**
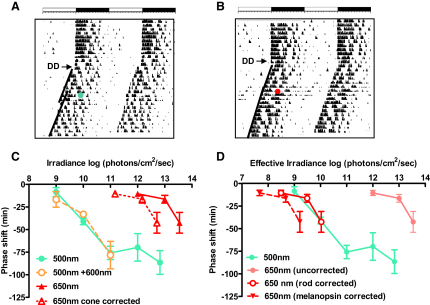
Rods Define Circadian Responses at Moderate Irradiances Representative double plotted actograms of wheel-running activity from the same *Opn1mw^R^* mouse exposed to 15 min pulses of 500 nm (10^11^ photons/cm^2^/s; A) or 650 nm (10^12^ photons/cm^2^/s; B) light at CT16 show a marked phase delay to the shorter, but not the longer, wavelength. The first 10–12 days show stable entrainment to a 12 hr:12 hr LD cycle (depicted as open/closed bars at the top of each panel), before release into DD at time indicated by arrow. Green/red circles represent the time of light exposure, with lines drawn through activity onsets before and after this pulse revealing the magnitude of the phase shift. (C) Irradiance response curves for phase delays elicited (mean ± SEM; n = 3–8) by 15 min pulses at CT16 using this paradigm in *Opn1mw^R^* mice reveal substantially reduced sensitivity to 650 nm light compared with 500 nm. Correcting for the relative sensitivity of cones at this wavelength was insufficient to account for the poor long-wavelength sensitivity. Responses to 500 nm were not altered by inclusion of 600 nm light at 10× higher irradiance (500+600 nm curve), confirming that cones make neither a strong stimulatory nor inhibitory contribution to this response. (D) Correcting the 650 nm irradiance response curve for the relative sensitivity of rods or melanopsin at this wavelength suggests that rods define the sensitivity of this response.

The same (M1) class of mRGC is responsible for routing rod and cone input to both the circadian clock and that portion of the pretectum responsible for the PLR (the oliviary pretectal nuclei shell; Baver et al., 2008; Güler et al., 2008). To establish whether cones contribute to circadian photoentrainment, we set out to determine whether that response showed an equivalent red shift in sensitivity in *Opn1mw^R^* mice. To this end, we constructed irradiance response curves for phase shifts in the free-running locomotor activity rhythm in *Opn1mw^R^* mice elicited by 15 min 500 or 650 nm stimuli presented in the early subjective night (CT16). As expected, 500 nm stimuli induced marked phase delays whose magnitude was irradiance dependent (Figure 3). Strikingly however, *Opn1mw^R^* mice showed very poor sensitivity to 650 nm. Significant phase delays were elicited only by bright (>10^13^ photons/cm^2^/s) stimuli, representing an increase in threshold irradiance of at least 1000× compared to 500 nm. This is much greater than that predicted for a response driven by red cones (Figure 3). Surprisingly, therefore, it seems that cones do not make a significant contribution to this assay of circadian photoentrainment even when presented with stimuli that lie within the photopic range.

Because there have been suggestions that cones might inhibit circadian responses to light (Figueiro et al., 2004), we continued to directly test this possibility by assessing responses to dichromatic 500 and 600 nm stimuli (see Experimental Procedures for a discussion of the potential significance of melanopsin's putative bistability for this treatment). Red cones are approximately equally sensitive to these two wavelengths whereas rods and melanopsin are ∼10× and ∼100× more sensitive, respectively, to the shorter wavelength (Figure 1B). Thus, including 600 nm light at a 10-fold excess increases the effective photon flux for cones by an order of magnitude but increases the activation of rods by only ∼2-fold, and melanopsin by even less. We found that inclusion of 600 nm had no discernable effect on the magnitude of phase shifts (Figure 3C), confirming that cones make neither a positive nor negative contribution to this response.

As further confirmation of this surprising result, we revisited the circadian phenotype of transgenic mice (*Opn4^−/−^ Gnat1^−/−^*) lacking critical elements of rod (Calvert et al., 2000) and melanopsin (Lucas et al., 2003) phototransduction cascades. Electroretinography confirms that these mice retain responses typical of cone, but not rod, photoreception (Figure S3). An earlier study of these “cone only” mice reported significant interindividual variability in entrainment to a 16 hr:8 hr LD cycle (Mrosovsky and Hattar, 2005), leaving the question of whether cones alone can reliably entrain the clock unanswered. Because that study used a single irradiance, we undertook a more systematic investigation of photoentrainment by testing wheel-running behavior under 12 hr:12 hr LD cycles at six different irradiances. We found that even at the brightest intensities (1 mW/cm^2^, >5000× brighter than threshold for wild-type mice), only two out of six *Opn4^−/−^ Gnat1^−/−^* animals unambiguously entrained with a phase angle equivalent to that observed in wild-type controls (Figure S4). At lower irradiances the number of *Opn4^−/−^ Gnat1^−/−^* mice entrained systematically declined, until all free-ran at 2 μW/cm^2^, an irradiance at least 10× brighter than that at which all wild-types entrain.

### Rod Contributions to Circadian Photoentrainment

Which photoreceptors drive circadian entrainment in the absence of significant cone input? Correcting the 650 nm phase shift irradiance response curve for the relative sensitivity of rods provided a good correspondence with the 500 nm curve, whereas correction for melanopsin did not (Figure 3D; F-test statistic comparing intercepts of curves fitted to the 500 and 650 nm data sets revealed significant differences when irradiances corrected for cone [p < 0.001] or melanopsin [p < 0.01], but not rod [p > 0.05], spectral sensitivity; slopes were similar at both wavelengths [p > 0.05], consistent with involvement of a single photopigment). This suggests that rods define circadian photoentrainment, even at irradiances at which cones provide the major contribution to the dark-adapted PLR (Figure 2) and pattern vision (Nathan et al., 2006).

**Figure 4:**
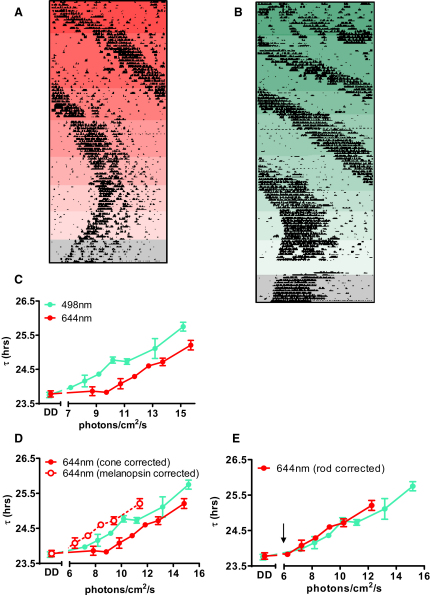
Responses to Constant Light Reveal a High Sensitivity Rod Input to the Circadian Clock (A and B) Representative actograms of wheel-running activity reveal irradiance-dependent increases in circadian period (τ) of *Opn1mw^R^* mice exposed to constant 644 nm (A) or 498 nm (B) light. Consecutive reductions in light intensity are depicted as reductions in background colored shading. The final 10 days of both records (gray shading) were collected in DD. (C) Irradiance response relationships for τ (estimated by periodogram analysis of activity records as shown in A and B) at 498 and 644 nm (mean ± SEM; n = 3–6), revealed substantially reduced sensitivity at the longer wavelength. (D) The difference in responsiveness to these two wavelengths could not be adequately accounted for by correcting for the relative sensitivity of either melanopsin or cones. (E) On the other hand, the curves became superimposed when corrected for rods. Arrow represents the estimated threshold for rod vision in mice (Nathan et al., 2006).

To investigate the photoreceptors regulating the clock in more detail, we turned to a different assay of circadian photoentrainment. When exposed to constant light, the period (τ) of mouse circadian rhythms lengthens according to Aschoff's rule (Daan and Pittendrigh, 1976). To determine the photoreceptors driving this response, we described its irradiance dependence in *Opn1mw^R^* mice exposed to either continuous mid- (498 nm) or long- (644 nm) wavelength light. Both wavelengths effectively lengthened τ (Figure 4). However, there was a marked decrease in sensitivity to the longer wavelength. Matching the irradiance response relationships for the relative sensitivity of red cones revealed that there was no irradiance at which τ was defined by cone photoreception (Figure 4D; F-test statistic; slopes at 498 nm and 644 nm similar, p > 0.05; intercepts significantly different [p < 0.001] when corrected for red cone spectral sensitivity). The curves were more equivalent when normalized for melanopsin sensitivity, but responses were clearly enhanced at 650 nm compared to that predicted for melanopsin, especially at lower irradiances (Figure 4C; F-test comparing intercepts, p < 0.001). By contrast, the curves became superimposed when corrected for rod sensitivity (Figure 4E; F-test comparing intercepts, p > 0.05), confirming that rod activity dominates this assay of photoentrainment, at least at low-moderate irradiances. The threshold for τ lengthening was around that reported for scotopic vision in mice (Nathan et al., 2006; Sampath et al., 2005), suggesting that mRGCs receive input from the highest-sensitivity rod pathways.

### Prior Light Exposure Limits the Cone Contribution to Pupillary Responses

Our pupillometry experiments confirm that cones can drive NIF responses in vivo (Figure 2). Furthermore, there is compelling published evidence that cone input reaches mRGCs (Dacey et al., 2005) and, indeed, clock neurons within the suprachiasmatic nuclei (Aggelopoulos and Meissl, 2000; Drouyer et al., 2007). Why then can we determine no discernable cone contribution to circadian photoentrainment? One possibility is that, thanks to light adaptation, the contribution of cones to pupillographic/electrophysiological responses elicited by transient stimuli is much greater than that to photoentrainment, which is, by its nature, defined by long-term light exposure (Dkhissi-Benyahya et al., 2007). Although cones are known to show rapid and extensive adaptation under extended illumination, they do attain a steady-state polarization, the magnitude of which is intensity dependent (Burkhardt, 1994; Normann and Perlman, 1979; Valeton and van Norren, 1983). Consequently, while there is an a priori expectation that light adaptation would impact cone input to the clock, this need not preclude cones from supporting photoentrainment. We therefore set out to determine empirically how cone contributions to NIF vision change under extended light exposure.

**Figure 5:**
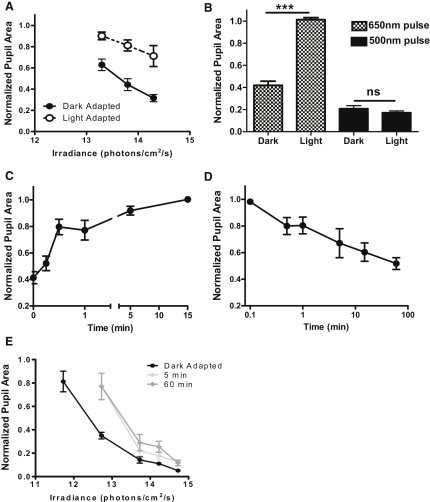
Cone Input to the PLR Is Reduced under Light-Adapted Conditions (A) Pre-exposure to 5 min 644 nm (10^13^ photons/cm^2^/s) substantially reduced pupillary responses (mean ± SEM; n = 6–7) to 10 s stimuli of equivalent or even higher irradiance (open circles) compared to those obtained following 1 hr of dark adaptation (closed circles). (B) Under more extensive adaptation (15 min 1.2 × 10^15^ photons/cm^2^/s; “Light”), pupil responses were completely absent throughout 30 s of exposure to a 650 nm test stimulus (2 × 10^14^ photons/cm^2^/s; hatched columns) capable of driving strong constriction under dark-adapted conditions (“Dark”). By contrast the response to an equivalent 500 nm (2 × 10^13^ photons/cm^2^/s; solid columns) test stimulus was unaffected. One-way ANOVA, p < 0.0001; selected post hoc tests with Tukey's correction shown, ^∗∗∗^p < 0.001; ns, p > 0.05. (C) The degree to which responses to the 30 s 650 nm test stimulus were inhibited was dependent upon the duration of prior light exposure (one-way ANOVA, p < 0.0001), although all exposures >15 s significantly impaired responses when compared with those of dark-adapted animals (Dunnett's post hoc comparisons, p < 0.01; n = 5–7). (D) Responsiveness to a 15 s 650 nm test stimulus (1.8 × 10^14^ photons/cm^2^/s) recovered over the course of 1 hr of dark adaptation (one-way ANOVA, p < 0.001; Dunnett's post hoc comparisons, p < 0.05, versus 0.1 min dark adaptation for times >1 min; n = 5–7). (E) Pre-exposure of *rd/rd cl* mice to 500 nm light (1.4 × 10^14^ photons/cm^2^/s) for either 5 or 60 min induced more classical light adaptation comprising a simple reduction in sensitivity to a subsequent 10 s 500 nm test pulse. All data points show mean ± SEM pupil area normalized to prestimulus condition.

We first explored the effects of prior treatment with 644 nm on subsequent cone-dependent pupillary responses in *Opn1mw^R^* mice. We found that 5 min of exposure to 10^13^ photons/cm^2^/s of 644 nm light greatly reduced pupil responses to a subsequent 650 nm test stimulus of equivalent irradiance (Figure 5A). Higher test irradiances drove larger constrictions, but the response magnitude was always substantially smaller than that elicited by an equivalent stimulus under dark-adapted conditions (Figure 5A). More extensive light exposure (15 min 1.2 × 10^15^ photons/cm^2^/s 644 nm) rendered the pupil entirely refractory to irradiances (10^14^ photons/cm^2^/s 650 nm) that drove large constrictions when dark-adapted (Figure 5B). To trace the kinetics of this effect, we assessed responses to the 650 nm test stimulus after 15 s, 30 s, 1 min, and 15 min exposure to 644 nm at 1.2 × 10^15^ photons/cm^2^/s. A significant decrease in response amplitude was observed at all exposure times bar the shortest, but only after 15 min were the animals entirely refractory to the 650 nm test pulse (Figure 5C). We traced dark adaptation as defined by the recovery of responses to the test stimulus following exposure to 15 min of 1.2 × 10^15^ photons/cm^2^/s 644 nm. This was also slow, with full recovery taking up to 1 hr (Figure 5D). Importantly, this did not reflect a general decrease in pupillary responsiveness because constrictions to 500 nm test stimuli survived even the brightest 644 nm pretreatment (Figure 5B). These data suggest that prior light exposure over timescales ranging from tens of seconds to tens of minutes reduces the ability of cones to regulate pupil size.

Our data imply that under light-adapted conditions photoreceptors other than cones play the predominant role in determining steady-state pupil size. There is evidence that melanopsin performs this function in primates (Gamlin et al., 2007). To determine whether this were also the case in mice, we finally tested melanopsin-dependent pupillary responses under light-adapted conditions using *rd/rd cl* mice. Pre-exposure to a bright adapting light (1.4 × 10^14^ photons/cm^2^/s 500 nm) for between 5 min and 1 hr induced a shift in sensitivity, but importantly did not reduce the ability of melanopsin to drive large pupil responses to irradiances equivalent to that of the adapting light (Figure 5E).

### Revealing Cone Input to the Circadian Clock

**Figure 6:**
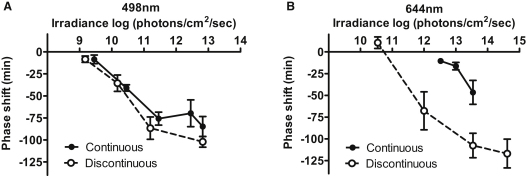
Temporal Contrast Reveals Cone Input to the Circadian Clock (A) Phase delays (mean ± SEM; n = 3–8) in *Opn1mw^R^* mice exposed to 500 nm at CT16 presented as 15 × 1 min pulses over 43 min are indistinguishable from those elicited by continuous 15 min exposure at this wavelength. (B) In contrast, at 650 nm the discontinuous stimuli were substantially more efficient than equiquantal continuous pulses at eliciting phase shifts (mean ± SEM; n = 5–9).

If the reduction in cone influence under light-adapted conditions revealed by the PLR were extended to other NIF responses, this could explain the inability of cones to support circadian photoentrainment. In that case, we reasoned that cone input to the clock might be revealed under conditions of very high temporal contrast. The circadian system is able to integrate photons over tens of minutes (Nelson and Takahashi, 1991), allowing discontinuous stimuli to be used to evoke phase shifts. Here we took advantage of this feature by presenting a total illumination time of 15 min as a series of 1 min pulses spread over 43 min, i.e. each separated by 2 min of darkness. This protocol drove phase shifts of equivalent magnitude to a continuous 15 min pulse in *rd/rd cl* mice (Figure S5). In *Opn1mw^R^* mice, we found that, at 500 nm, responses to the continuous and discontinuous stimuli were indistinguishable (Figure 6A). On the other hand, the introduction of discontinuity greatly enhanced phase shifting responses to long-wavelength (644 nm) light in this genotype (Figure 6B). This red shift in the spectral sensitivity of circadian photoentrainment confirms that high temporal contrast can effectively reveal cone input to the circadian clock.

## Discussion

Using *Opn1mw^R^* mice has allowed us to describe distinct rod, cone, and melanopsin contributions to mRGC-driven behaviors by comparing responses to medium- and long-wavelength light. This approach has significant advantages over alternative strategies based upon targeted disruption of specific photoreceptor classes. First, because our conclusions do not depend on comparisons against a wild-type control group, they are less prone to genetic and environmental confounds. Second, because physiological and behavioral analyses confirm that conventional visual pathways are functional in *Opn1mw^R^* mice (Jacobs et al., 2007; Smallwood et al., 2003), experiments with these animals allow us to explore the process of irradiance measurement in an organism with an intact visual system. For these reasons we argue that experiments in this genotype are likely to provide the most accurate and complete picture possible of rodent irradiance measurement. They do not however reveal what each photoreceptor can achieve in isolation. This somewhat different question is covered by studies here and elsewhere with “cone only” (*Gnat1^−/−^ Opn4^−/−^*), “melanopsin only” (*rd/rd cl*), and “melanopsin-less” (*Opn4^−/−^*) genotypes (Hattar et al., 2003; Lucas et al., 2001, 2003; Mrosovsky and Hattar, 2005; Panda et al., 2002, 2003; Ruby et al., 2002). The obvious omission in this latter panel is a “rod only” mouse, which would reveal the absolute limits of rod influence. Those studies are currently under way. Finally, an important area of future work will be to address any distinct role of s-cone pathways (Haverkamp et al., 2005) in NIF vision.

**Figure 7:**
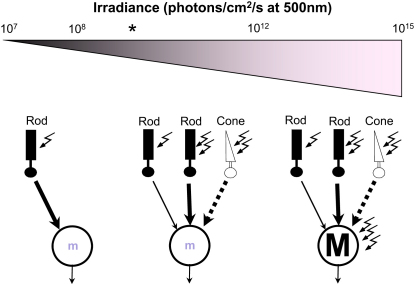
These Data Suggest an Irradiance Measurement System with Four Distinct Inputs At very low irradiances responses rely solely on rods, probably signaling via the highest-sensitivity rod visual pathways. Over a broad range of moderate irradiances, lower-sensitivity rod and cone pathways take over. Under dark-adapted conditions cones dominate NIF responses, but their influence is reduced under light adaptation. This suggests that under most field conditions cones are restricted to contributing high-frequency modulation of pupil size with rather little influence on systems such as the circadian clock that integrate light signals over prolonged timescales. Under these circumstances, currently undefined rod pathways play the major role. Melanopsin phototransduction has low sensitivity, allowing it to encode high irradiances. Our data suggest thresholds of ∼10^7^, 10^8^, and 10^12^ 500 nm photons/cm^2^/s for rod, cone, and melanopsin inputs, respectively. Asterisk depicts approximate threshold for murine cone-based vision for reference (Nathan et al., 2006).

Our findings with *Opn1mw^R^* mice suggest at least four distinct contributions to irradiance measurement (Figure 7). The first is from a very high-sensitivity rod pathway. The threshold for light-dependent increases in circadian period revealed here (Figure 4) is very close to that reported for rod-based vision in mice (Nathan et al., 2006; Sampath et al., 2005). This is consistent with reports from a number of species that very dim light can influence circadian entrainment (Bachleitner et al., 2007; Evans et al., 2009). In the context of the mammalian retina, it implies that the highest-sensitivity rod pathway (comprising rod bipolar and AII amacrine cells; Deans et al., 2002) can influence mRGCs.

Surprisingly, we did not observe an equivalent rod-dependent phase to the dark-adapted PLR. The literature suggests that this very high-sensitivity rod signal is present in pupillary afferents because, working with anesthetized mice, Pennesi et al. (1998) reported pupil constriction at extremely low-light intensities (<100× lower than the threshold for a reproducible response in our experiments). Anesthesia drives partial miosis (presumably reflecting alterations in autonomic activity), and the most parsimonious explanation for our failure to detect this dim light response is that intact sympathetic input to the iris in unanasthetized animals antagonizes rod-dependent constriction, rendering it immeasurable. The degree to which this high-sensitivity rod signal actually constricts the pupil under “field” conditions remains moot.

The high-sensitivity rod pathway saturates at relatively low light intensities (Pang et al., 2004). One might therefore expect a strong cone influence to become apparent at moderate irradiances. This is exactly what we observe for the dark-adapted PLR, with cone photoreception defining pupil size between 10^8^ and 10^11^ photons/cm^2^/s (Figure 2). Surprisingly, however, there was no equivalent cone contribution to circadian phase shifts within this irradiance range (Figure 3). This finding contradicts a published model of cone inputs to the mouse clock (Dkhissi-Benyahya et al., 2007) but is consistent with some of the findings upon which it is based (specifically that phase shifts induced by 480 nm light are normal in the absence of m-cones), as well as other reports that circadian responses are not affected by cone degeneration (Freedman et al., 1999).

We present data from both pupillary and circadian experiments suggesting that an important factor limiting cone influence on mRGCs is light adaptation. Thus, prior light exposure strongly reduces the magnitude of cone-dependent pupillary constriction, whereas cone-dependent phase shifts can be induced using intermittent light exposure. An important unanswered question is the degree to which this simply reflects adaptation in cone phototransduction. When cones are presented with an extended light step, their membrane potential “relaxes” over time to a level around half the peak hyperpolarization (Burkhardt, 1994; Normann and Perlman, 1979). In primate cones, this steady-state response persists for at least tens of minutes (Burkhardt, 1994; Normann and Perlman, 1979). This manifestation of photoreceptor adaptation would be expected to impact cone input to NIF responses. However, its magnitude would appear too small to explain, for example, our observation that there is essentially no cone influence on circadian phase shifts induced by 15 min stimuli at any irradiance. It seems likely therefore that changes in the voltage gain of the synaptic pathway linking cones to mRGCs make a strong contribution to adaptation in cone input to the NIF system. This highlights the need for a greater understanding of the behavior of neural circuits (including the unusual cone ON bipolar cells; Dumitrescu et al., 2009; Hoshi et al., 2009) upstream of mRGCs. Whatever its origin, it is noteworthy that light adaptation, which acts to greatly increase the range of lighting conditions over which cones can support pattern vision, has quite the opposite effect for NIF responses.

The limited ability of cones to drive responses to moderate irradiances under light-adapted conditions raises the question of which photoreceptor takes over under these conditions. Our analysis of the spectral sensitivity of both increases in τ and circadian phase shifts over this moderate irradiance range in *Opn1mw^R^* mice implicate rods (Figures 3 and 4). While this finding may be surprising because these irradiances are clearly within the sensitivity range of cones, it is consistent with reports that the wild-type rodent clock shows peak sensitivity around 500 nm (Provencio and Foster, 1995; Takahashi et al., 1984; Yoshimura and Ebihara, 1996). Moreover, rods have been shown to support visual discrimination in coneless mice over similar light intensities (Nathan et al., 2006; Williams et al., 2005), which fall comfortably short of those needed to induce significant rod bleach (see Experimental Procedures).

It will be interesting to determine the route by which this relatively low-sensitivity rod signal reaches mRGCs. The accepted pathway for rod signal transfer under mesopic/photopic conditions is via gap junctions to cones and thence through the cone bipolar cell population to RGCs. If rod input to mRGCs employs this pathway it is hard to envisage how it could avoid any network adaptation in the cone input to mRGCs (see above). Alternative possibilities include direct connections from rods to a subset of cone ON bipolars (Abd-El-Barr et al., 2009; Pang et al., 2004), or direct input from rod bipolar cells to mRGCs (Østergaard et al., 2007).

The final component of irradiance measurement, active at the brightest irradiances, is melanopsin phototransduction. Our pupillary data suggest that the melanopsin threshold in vivo is around 1000× lower than that of cone-based vision. However, *rd/rd cl* mice show circadian phase shifts to 15 min stimuli at least 10-fold dimmer than this (Hattar et al., 2003), suggesting that melanopsin phototransduction may be active at surprisingly dim irradiances. In any event, our data suggest that under most daylight conditions, melanopsin would be the primary influence on mRGC activity.

Because the same (M1) subtype of mRGC drives both circadian and pupillary responses studied here (Baver et al., 2008; Güler et al., 2008), our findings may not hold for aspects of NIF vision reliant on other mRGC classes. Leaving aside this limitation, our data suggest a relatively simple segregation of photoreceptor inputs to NIF vision under field conditions. They predict that rods play the predominant role in driving responses at night and around dawn/dusk with melanopsin taking over throughout most daylight. Light adaptation would limit cone influence under most conditions. However, this may allow cones to encode a somewhat different aspect of the light environment. Thus, the relatively sluggish adaptation we record here would, in effect, introduce a high-pass filter, reducing the influence of the tonic component of cone activity under continuous illumination in favor of more phasic responses to sudden changes in irradiance. This would free cones to provide higher-frequency modulation of pupil size (Gamlin et al., 2007; Young and Kimura, 2008). The circadian clock, because of its long integration time for photic information, would be relatively refractory to these transient cone signals except under conditions of high temporal contrast (Figure 6).

## Experimental Procedures

Experiments were in compliance with relevant laws and institutional guidelines at the University of Manchester and Johns Hopkins University. Founders for our *Opn1mw^R^* (transgenic allele previously referred to as *R* [Smallwood et al., 2003] and *L* [Jacobs et al., 2007]; *Opn1mw^tm1(LW)JN^* according to the rules of the International Committee on Standardized Genetic Nomenclature for Mice) colony were a generous gift of J. Nathans (Johns Hopkins Medical Institute). In females heterozygous for the knockin allele, X-inactivation ensures separate populations of mid- and long-wavelength sensitive cones. However, in these experiments we used exclusively hemizygous males (referred to here as *Opn1mw^R^*) or female homozygous knockins (*Opn1mw^R/R^*), neither of which retain a copy of the native mouse m-cone opsin and thus lack mid-wavelength sensitive cones.

### Pupillometry

Pupillometry was conducted as previously described (Lucas et al., 2003) on unanesthetized adult (50–190 days) mice. Animals were stably entrained to a 12 hr:12 hr LD cycle (white fluorescent source, ∼180 lux) and recordings were restricted to between 4 and 7 hr after lights on. All experiments were preceded by 1 hr of dark adaptation. Pupillary responses were elicited with Ganzfeld light stimuli (Xe arc source, filtered with neutral density and monochromatic interference filters, half bandwidth ≤10 nm) applied to one eye, previously dilated with 0.1% atropine (except for studies of light adaptation in which no midriatic was employed), allowing consensual pupil constriction to be recorded with a CCD camera. Except when otherwise indicated, 10 s of darkness separated pretreatment and test stimuli in all light adaptation experiments. Pupil area was measured using analysis software written in Matlab and expressed relative to its size in the 3 s prior to light onset. For adaptation experiments, pretreatment was applied in a specialized chamber with full internal reflectance using an LED source (Philips LumiLED; λ_max_ 498 or 644 nm, half-bandwidth ≤37 nm). There was a standard 10 s gap between removal from this adapting light and application of the test stimulus during which the animals were in darkness.

### Circadian Photoentrainment

Circadian rhythms were assessed by monitoring wheel-running activity using standard methods. Briefly, male mice were singly housed with free access to a running wheel, revolutions of which were monitored using either the Chronobiology Kit (Stanford Software Systems, Santa Cruz, CA) or Clocklab (Actimetrix, Wilmette, IL). To determine the limits of entrainment for *Opn4^−/−^ Gnat1^−/−^* mice, animals were exposed to a 12 hr:12 hr LD cycle. One group of mice were exposed to 1 m W/cm^2^ (Philips Daylight deluxe fluorescent source; corresponds to 500 lux) for 21 days before being released into constant darkness (DD). To explore the irradiance dependence of entrainment of *Opn4^−/−^ Gnat1^−/−^* mice, a second group of animals were first exposed to the same lighting regime at 235 μW/cm^2^, followed by log unit decreases in irradiance every 2 weeks down to 0.2 μW/cm^2^. The irradiance at which animals free-run was defined as the point at which the period of their activity rhythm differed from 24 hr. To measure phase shifts, mice were first housed for at least 14 days under a 12 hr:12 hr LD cycle (white fluorescent light, ∼180 lux) before being released into DD. Light pulses at CT16 (4 hr after activity onset) were administered after 7 days in DD and animals were left for a further 10 days. Regression lines through the time of activity onset before and after the light pulse were used to calculate the phase shift. Light stimuli were applied in a specialized chamber with full internal reflectance using light from either a Xe arc source (filtered with neutral density and monochromatic interference filters, half bandwidth ≤10 nm) or, when higher intensities were required, an LED source (Philips LumiLED; λ_max_ 498 or 644 nm, half bandwidth ≤37 nm). This apparatus allows near full-field illumination, but some complexity in the visual scene is unavoidable because animals are freely moving. For constant light experiments, diffuse near monochromatic light (λ_max_ 498 or 644 nm, half bandwidth ≤37 nm) was applied using custom-built LED arrays (Philips LumiLED) supported above the home cage. In the case of LED sources, the effective photon flux for red cone, rod, or melanopsin photoreceptors was determined by calculating the photon flux at each wavelength, corrected according to the spectral efficiency function for that pigment, and integrating across wavelengths.

### Light Measurements

Light measurements employed an optical power meter (Macam Photometrics, Livingstone, UK) and a spectrometer (Ocean Optics Inc., Dunedin, FL) as appropriate. In the case of near monochromatic stimuli, we were able to convert these power measurements to photon flux (ϕ_p_ in photons/cm^2^/s) according to the formula: ϕ_p_ = P × λ × 5.03 × 10^15^; where p = power in W and λ = wavelength.

To facilitate comparisons with other publications, these can also be expressed as scotopic cd/m^2^ using the correction factor suggested by Lyubarsky et al. (2004) adjusted according to the rod opsin spectral efficiency curve (1 cd/m^2^ = 3.7 × 10^11^ photons/cm2/s for stimuli near 500 nm; 1.9 × 10^12^ photons/cm2/s for stimuli near 600 nm; and 1.2 × 10^15^ photons/cm2/s for stimuli near 650 nm).

In view of the evidence that rods drive circadian responses at relatively high irradiances (Figures 3 and 4), it is interesting also to relate the light intensities used in these studies to those required for significant rod opsin bleach. Alpern (1971) reported little rod opsin bleach in humans under steady exposure to retinal illuminance <10^3^ scotopic td. Lyubarsky et al. (2004) estimate 13× to 20× as many rod photoisomerizations per troland in mice compared with humans, largely because of the difference in retinal area. On this basis the lower limit for significant rod bleach in mouse would be ∼50 scotopic td. To convert this figure to corneal luminance, we need to correct for pupil area. This is unknown for our circadian experiments, but a fairly dilated pupil (area = 3.2 mm^2^) can be assumed for simplicity, while acknowledging that this may overestimate retinal illumination by >10× if the pupil were fully constricted. This gives a conservative estimate of 16 cd/m^2^ (or 6 × 10^12^ photons/cm^2^/s for stimuli near 500 nm) as a lower limit for significant rod opsin bleach in our experiments. Fifty percent bleach would then require irradiances at least 10× greater than this.

Note that these measurements and conversions will provide an accurate approximation of corneal irradiance in the phase shifting and pupillometry experiments for which a Ganzfeld stimulus was employed, but less so for the LL study in which light was applied to the subject's home cage.

### A Note on the Significance of Melanopsin's Putative Bistability for Our Choice of Light Treatments

Indications that melanopsin may employ an intrinsic photoreversal mechanism for bleach recovery raise the possibility that its photosensitivity is increased by prior exposure to long-wavelength light. A consensus on this question has not been reached (see Rollag, 2008 for discussion), and our work was not designed to contribute to that debate. Most of the experiments reported here have employed near monochromatic stimuli for which melanopsin's sensitivity can be well described by an opsin nomogram λ_max_ ≈ 480 nm (Berson et al., 2002; Hattar et al., 2003; Lucas et al., 2001), and this is the model upon which we have based our interpretation. Melanopsin's bistability could be more of a consideration in the phase shifting responses elicited by dichromatic 500 nm and 600 nm light (Figure 3A), but because we saw no significant divergence from responses to 500 nm alone this seems not to be the case. Finally, while pigment bleach could be one mechanism of light adaptation driving the shift in pupil sensitivity of *rd/rd cl* mice following pretreatment with 500 nm (Figure 5E), we do not attempt to distinguish between this and other explanations for this finding.

## Note Added in Proof

While this manuscript was under review, two papers were published exploring the photoreceptive origins of the human pupil light reflex (McDougal and Gamlin, 2010; Tsujimura et al., 2010). Their findings are broadly consistent with the model in Figure 7. McDougal and Douglas also coined the phrase “winner takes all” to describe the summation of inner and outer retinal signals in human pupillomotor afferents.
