# Supplementary material for: Distinct Contributions of Rod, Cone, and Melanopsin Photoreceptors to Encoding Irradiance
Source: Neuron. 2010 May 13;66(3):417–28. doi: 10.1016/j.neuron.2010.04.037 (PMC2875410; doi:10.1016/j.neuron.2010.04.037)
Supplement: Document S1. Supplemental Figures [file mmc1.pdf]

Neuron, volume *66*

Supplemental Information

Distinct Contributions of Rod, Cone, and Melanopsin Photoreceptors to Encoding Irradiance

Gurprit S. Lall, Victoria L. Revell, Hiroshi Momiji, Jazi Al Enezi, Cara M. Altimus, Ali D. Güler, Carlos Aguilar, Morven A. Cameron, Susan Allender, Mark W. Hankins, and Robert J. Lucas

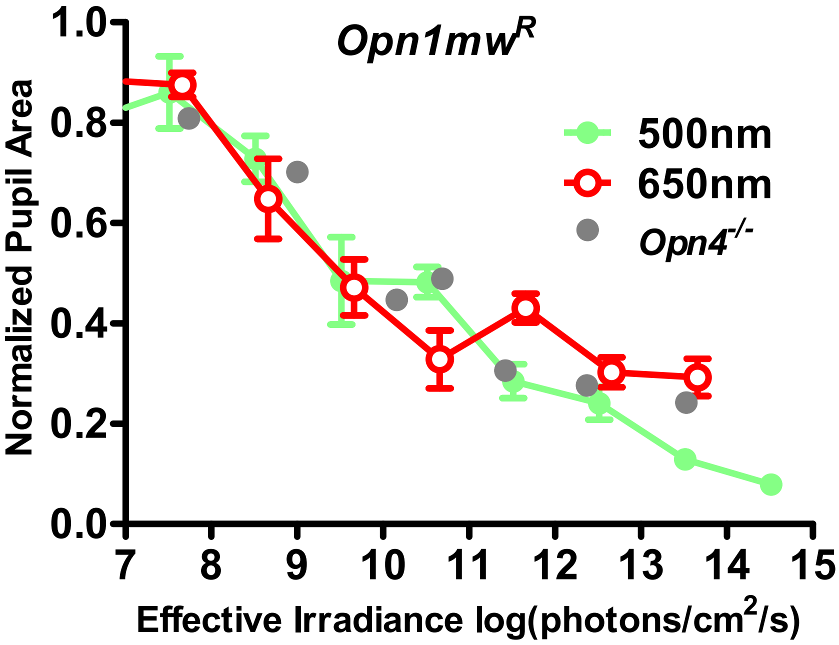

Figure S1: The response of *Opn1mw*^{R} mice to 650nm but not 500nm light (mean±SEM n=4-12 replotted from Figure 2) is qualitatively similar to historical data from *Opn4*$^{-/-}$ animals (mean response for n=5-8 mice replotted from Lucas *et al (*2003) Diminished pupillary light reflex at high irradiances in melanopsin-knockout mice. **Science** 299:245-247).

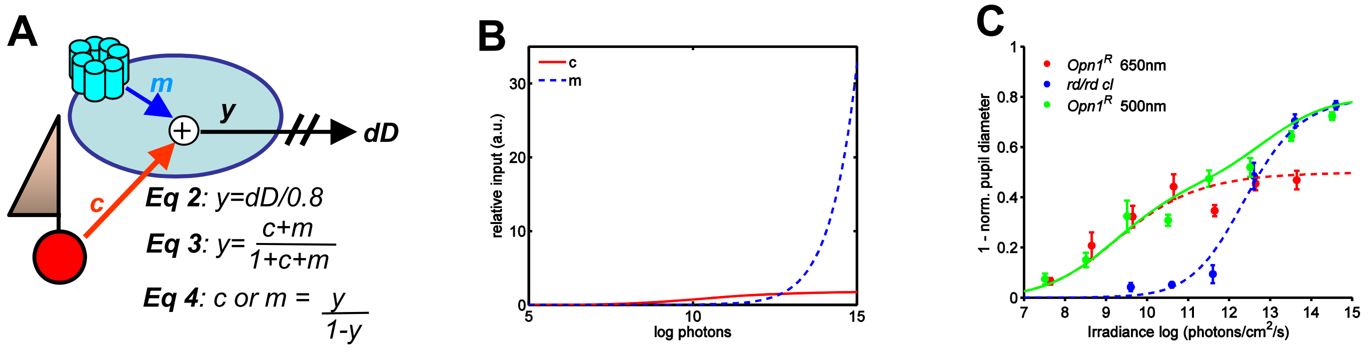

Figure S2: A quantitative model for integrating cone and melanopsin signals within irradiance detection pathways. We set out to determine a method by which the irradiance response relationship for *Opn1mw*^{R} at 500nm (to which both cones and melanopsin contribute) could be approximated from the responses driven by cones (approximated by the *Opn1mw*^{R} 650nm response) or melanopsin (*rd/rd cl)* alone. We started, by fitting the data in Figure 2B (i.e. after normalising for red cone sensitivity) and C with a monotonic function of the form:

$$D(I) = D_{\min} + (1-D_{\min}) \frac{I_0^n}{I_0^n + I^n} \qquad \text{Eq. (1)}$$

Where D is the normalized pupil diameter, calculated from the normalized pupil area and used because it is thought to have a simpler relationship with irradiance than does area. We then applied these data to a simple conceptual model (**A**) in which mRGCs encode irradiance as a parameter (*y*) that is the product of intrinsic (melanopsin; *m*) and extrinsic (cone; *c*) inputs. For simplicity, we assume a linear relationship between *y* and the degree of constriction (dD; defined as 1 – *D*), and a nominal value of 1 for the magnitude of *y* required to drive a saturating constriction (normalized area ~0.03, corresponding to dD=0.8). Thus *y* can be estimated as *y*=dD/0.8 (Eq. 2) at any irradiance/wavelength from the data presented in Figure 2A. To account for the notable experimental observation that the melanopsin alone can drive a saturating pupil constriction, *y* was modeled with a minimal Hill function:

$$y = \frac{x}{1+x} = \frac{c+m}{1+c+m} \qquad \text{Eq. (3)}$$

Eq. (3) can be rewritten for *x* as a function of *y* as follows.

$$x = \frac{y}{1 - y} \qquad \text{Eq. (4)}$$

B. Therefore, by using Eq. (4), cone input (*c*) is estimated from the mRGC signal (*y* = *dD*/0.8) of *Opn1mw*^{R} 650nm, for which melanopsin input (*m*) is 0, while *m* is estimated from *y* of *rd/rd cl* 500nm, for which *c* is 0. **C.** When applied to Eq. 3 these estimates of *c* and *m* provided a good approximation of experimentally defined pupil responses for *Opn1mw*^{R} at 500nm (at which both cone and melanopsin are functional). The large discrepancy in the magnitude of *c* and *m* at higher irradiances (**B**) implies that the ability of melanopsin to influence mRGC activity over these timeframes far exceeds that of cones.

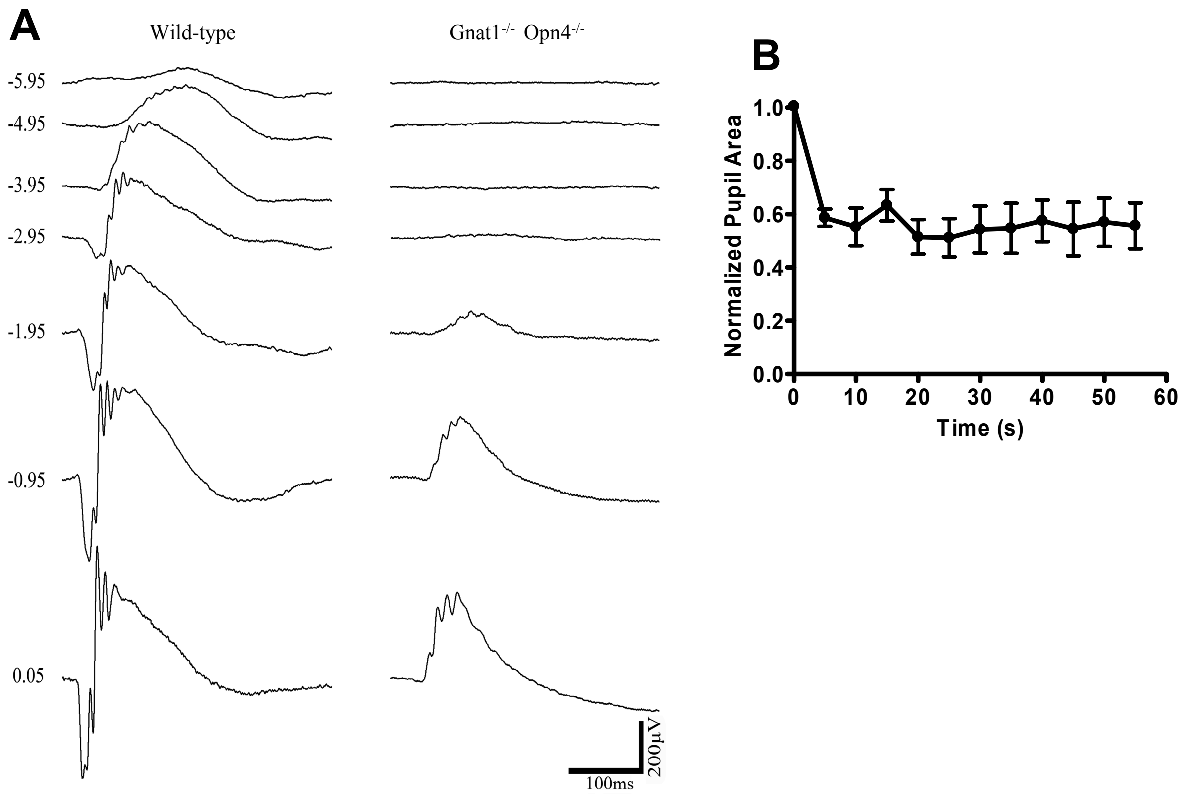

Figure S3: Electroretinogram and pupillary responses in *Opn4*$^{-/-}$ *Gnat1*$^{-/-}$ mice. **A.** Representative flash ERG traces from dark adapted *Opn4*$^{-/-}$ *Gnat1*$^{-/-}$ (right) and wild type (left) mice reveal selective loss of dim (<-2.95 log W/m^2; <-0.48 log cd-s/m^2) but not bright light responses in the transgenic animals confirming the presence of cone but not rod pathways. Figures to the left are stimulus irradiance in log W/m^2. Electroretinography was undertaken under ketamine (70mg/kg) and xylazine (7mg/kg) anaesthesia as previously described (Barnard et al., 2006). Mice were long-term dark adapted (>12hr) prior to recording scotopic ERGs using 15ms ganzfled flash stimuli (Xe arc source; Cairn Research Ltd., Faversham, UK) were applied at corneal irradiances in the range -5.95 to 0.05 log W/m^2 (-3.48 to 0.05 log cd-s/m^2) Inter-stimulus interval (ISI) was 1.5s at the dimmest intensities and was increased, proportionally with irradiance, to 30s at the brightest intensities. Similarly, the number of repetitions decreased from 30 to 6 as the stimulus intensity was increased. Signals were band-pass filtered 0.5 to 200Hz, and digitized at a sampling rate 2kHz. **B**. The ability of cones to drive NIF responses is confirmed by a marked and sustained reduction in pupil size (mean±SEM; n=4) to a 60s 480nm light stimulus (5.7x10^{14} photons/cm^2/sec; switched on at time 0) in dark adapted *Opn4*$^{-/-}$ *Gnat1*$^{-/-}$ mice.

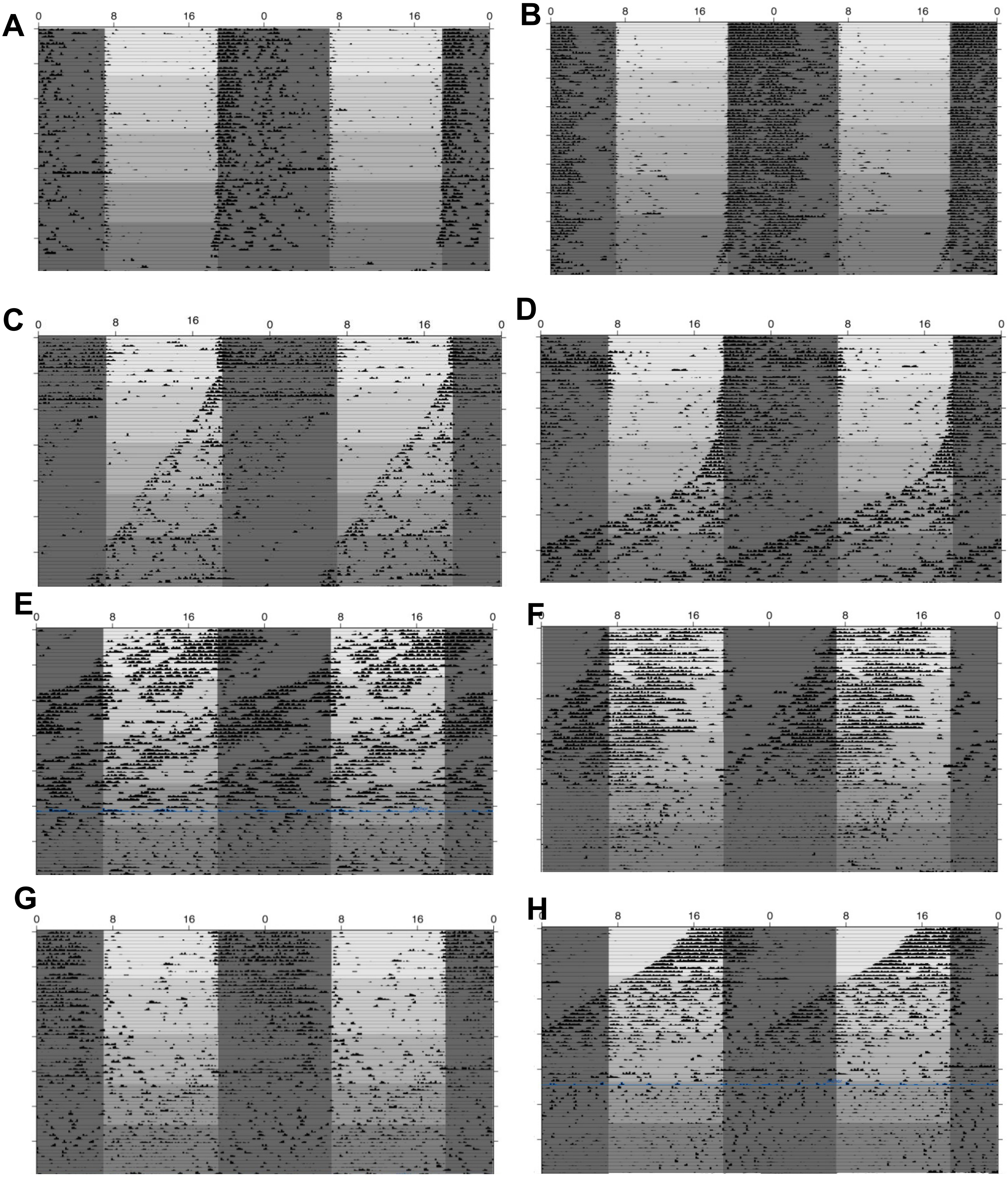
A
B
C
D
E
F
G
H
0 8 16 0 8 16 0

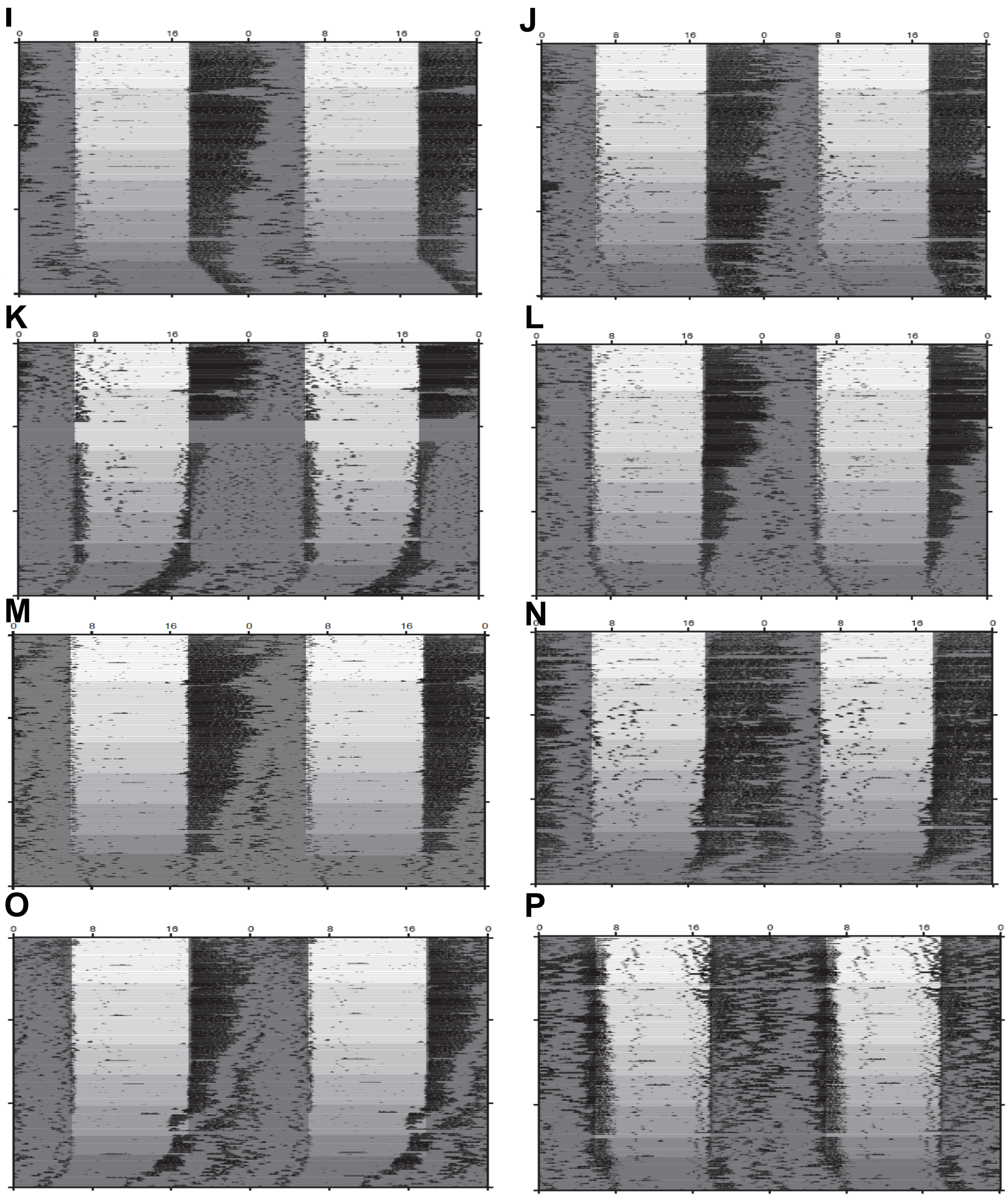

I
J
K
L
M
N
O
P
0
8
16
0
8
16
0

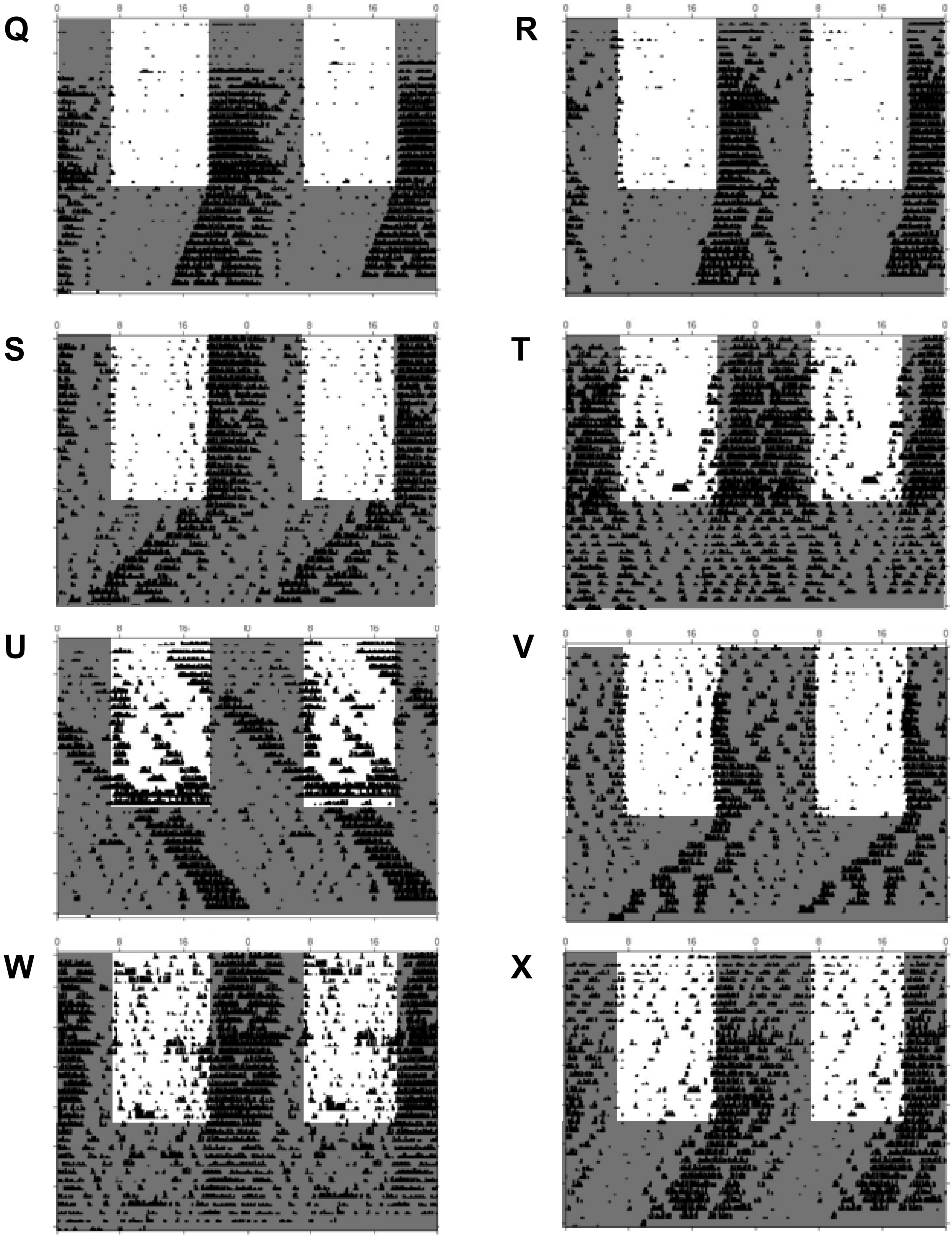
Q
R
S
T
U
V
W
X
0 8 16 0 8 16 0

Figure S4: *Opn4$^{-/-}$ Gnat1$^{-/-}$* mice show inconsistent circadian photoentrainment. Representative double plotted actograms from wild type (**A**&**B**) and *Opn4$^{-/-}$ Gnat1$^{-/-}$* (**C**-**H**) mice exposed to a 12h:12h LD cycle (dark phase shown as intense background shading) in which the irradiance of the light phase started at 235μW/cm^2 (fluorescent white source) and was reduced by a factor of 10 at two weekly intervals (depicted as increases in shading). At 235μW/cm^2 half (3/6) of *Opn4$^{-/-}$ Gnat1$^{-/-}$* mice showed clear free-run (period<24h), and only 1/6 entrained down to 23.5μW/cm^2. By contrast, all wild type mice entrained to as little as 0.235μW/cm^2. Similar records from *Opn4$^{-/-}$* (**I-M**) confirm that mice of this genotype show similar entrainment as wild types (**N-P**) at a range of irradiances from 235-0.0235μW/cm^2 (irradiance reduced by at times indicated by increases in shading x10 until finally released into DD) indicating that this was not a reflection of melanopsin loss. To determine whether *Opn4$^{-/-}$ Gnat1$^{-/-}$* entrainment would be more normal at higher irradiances, we subjected a further group of *Opn4$^{-/-}$ Gnat1$^{-/-}$* mice to a 12:12 LD cycle at an irradiance of 1mW/cm^2. Double-plotted actograms (periods of darkness indicated by shading) confirm that wild types (**Q** & **R**, representative of 4 such records) entrain well to this cycle until released into free-run in constant darkness. By contrast, even at this highest light intensity only a minority of *Opn4$^{-/-}$ Gnat1$^{-/-}$* mice (**S**-**X**) showed clear 'wild type-like' entrainment with an appropriate phase angle (**V**, **X**). The remainder free-ran (**U**), entrained with a strong positive phase angle (**S**) or exhibited $t \approx$ 24h, making an assessment of entrainment difficult (**T**&**W**). These findings are thus consistent with the conclusion drawn from *Opn1mw^R* experiments that while cones do provide light input to the clock, their ability to support entrainment is limited.

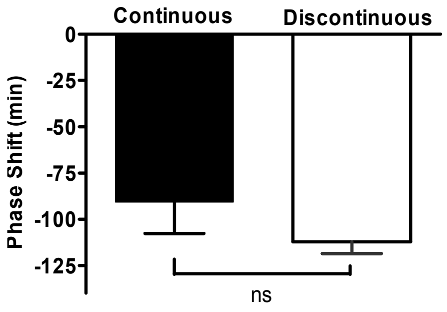

Figure S5: *rd/rd cl* mice show equivalent phase shifts (mean±SEM; n=4; paired t-test $p>0.05$) to 1.5×10^{13} photons/cm^2/sec of 500nm presented either as a single 15min (continuous) stimulus (filled bar) or as 15x1min (discontinuous) pulses spread over 43min (empty bar).
